# Prioritizing Measures of Digital Patient Engagement: A Delphi Expert Panel Study

**DOI:** 10.2196/jmir.4778

**Published:** 2017-05-26

**Authors:** Lynn A Garvin, Steven R Simon

**Affiliations:** ^1^ Department of Health Law, Policy and Management School of Public Health Boston University Boston, MA United States; ^2^ Geriatrics and Extended Care Service Veterans Affairs Boston Healthcare System Boston, MA United States; ^3^ The Center for Healthcare Organization and Implementation Research Veterans Affairs Boston Healthcare System Boston, MA United States; ^4^ Division of General Medicine and Primary Care Brigham and Women's Hospital Boston, MA United States

**Keywords:** patient reported outcome measures, patient engagement, patient-centered care, personal health record, health information technology, veterans health

## Abstract

**Background:**

Establishing a validated scale of patient engagement through use of information technology (ie, digital patient engagement) is the first step to understanding its role in health and health care quality, outcomes, and efficient implementation by health care providers and systems.

**Objective:**

The aim of this study was to develop and prioritize measures of digital patient engagement based on patients’ use of the US Department of Veterans Affairs (VA)’s MyHealtheVet (MHV) portal, focusing on the MHV/Blue Button and Secure Messaging functions.

**Methods:**

We aligned two models from the information systems and organizational behavior literatures to create a theory-based model of digital patient engagement. On the basis of this model, we conducted ten key informant interviews to identify potential measures from existing VA studies and consolidated the measures. We then conducted three rounds of modified Delphi rating by 12 national eHealth experts via Web-based surveys to prioritize the measures.

**Results:**

All 12 experts completed the study’s three rounds of modified Delphi ratings, resulting in two sets of final candidate measures representing digital patient engagement for Secure Messaging (58 measures) and MHV/Blue Button (71 measures). These measure sets map to Donabedian’s three types of quality measures: (1) antecedents (eg, patient demographics); (2) processes (eg, a novel measure of Web-based care quality); and (3) outcomes (eg, patient engagement).

**Conclusions:**

This national expert panel study using a modified Delphi technique prioritized candidate measures to assess digital patient engagement through patients’ use of VA’s My HealtheVet portal. The process yielded two robust measures sets prepared for future piloting and validation in surveys among Veterans.

## Introduction

Patient portals are Web-based platforms that provide patients with access to health information and elements of their medical record and equip them with tools to interact with their clinical teams [1]. Numerous studies have assessed the adoption of patient portals, including the factors that predispose patients to adoption as well as the barriers and challenges they face [2-8]. Moving beyond adoption, measuring the nature and extent of patients’ use of portal tools is a priority for the US Department of Veterans Affairs (VA) and other health systems. This emerging focus on measuring *digital patient engagement* [9] follows organically from—and is inextricably linked with—measuring *clinician meaningful use* of health information technology (HIT) as supported by the US Centers for Medicare & Medicaid Services’s “Meaningful Use” rules [10]. However, to date, neither VA nor any other US health system has established nationally validated measures of digital patient engagement to provide an indication of the extent to which patients are genuinely using portal-based tools and the degree to which those tools are engaging patients with their health care. The main objective of this study was therefore to develop and prioritize measures of patients’ experience using functions of a patient portal, with specific attention to the degree to which their experience using those functions promotes engagement with their health care team. To achieve this objective, we used a modified Delphi panel. This systematic approach aggregated experts’ opinions and perceptions of which measures would be most valuable and appropriate for assessing Veterans’ use of Blue Button and Secure Messaging, two salient features of My HealtheVet, VA’s patient portal. [11]. Our study focused on the Blue Button function, which allows Veterans to download their personal health record, and the Secure Messaging function, which enables Veterans to communicate via secure email with their health care team.

## Methods

This study involved a sequence of three phases: (1) literature review; (2) key informant interviews; and (3) Delphi panel process. For the literature review, we sought to identify prior work that would enable us to design a theory-based model for the study; as such, we researched three literature streams of established frameworks and validated scales of patients': (1) health and health care, (2) use of IT or HIT, and (3) relations with providers and health care systems. This literature review indicated that existing models of patient engagement and of technology adoption did not sufficiently overlap or integrate with each other to provide a framework for measuring patients’ engagement in their health and health care through technology.

We therefore defined *digital patient engagement* as the value that a patient (or family member or caregiver) assigns to the accrued experience with and results of using a system feature or service plus the expectation of similar future experience and results. On the basis of our theory-based definition, we aligned two established models: (1) Technology Acceptance Model [12] to reflect the functional dimension and (2) Relational Coordination Model to reflect the patient-provider dimension of digital patient engagement [13]. The resulting model included both patient engagement and intent to use and recommend the technology as outcomes reflecting digital patient engagement.

**Figure 1:**
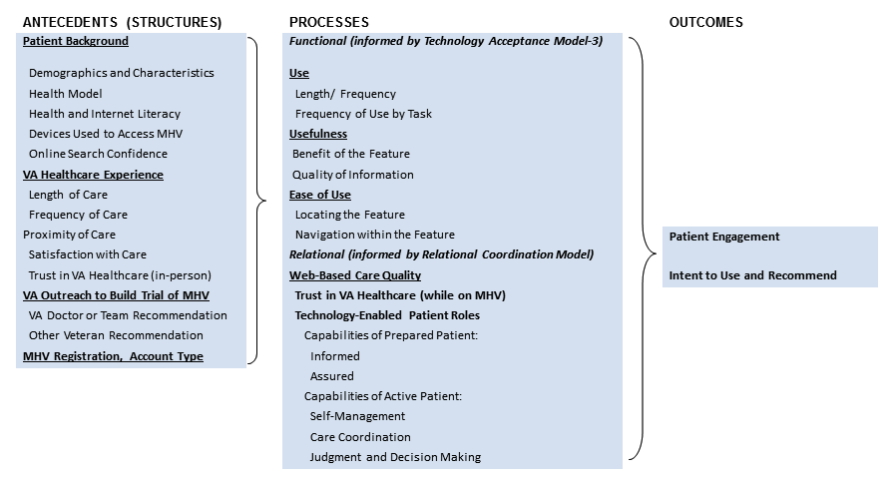
Digital patient engagement model.

Our resulting theory-based model is shown in Figure 1. The “account type” mentioned in the figure refers to the type of My HealtheVet account the user possesses. Account types included basic, which provided online access to general health information; advanced, which included access to the Blue Button and the ability to view other elements of the personal health record; and premium, which included all of the advanced features but added secure messaging capability.

For the second phase of the study, in late 2012, we conducted semistructured key informant telephone interviews with principal investigators of all current VA-funded MyHealtheVet (MHV) studies (N=10) to identify existing measures of MHV adoption and use. The ten participants were identified through communication with the MHV Program Office and with researchers in the field. One or both of the authors conducted telephone interviews with each participant.

Following interviews, we conducted the third phase of the study, a modified Delphi process. We followed the methods of previously published studies [14-21] and tailored them to the objectives herein. The modified Delphi technique is an intensive, iterative approach to elicit and refine experts’ opinions on novel conceptual fields with the goals of gaining consensus on candidate measures and evolving the framework. From the literature review and in-depth interviews, we generated a set of candidate measures to be considered by the Delphi panel, with the goal of identifying measures of use of the Blue Button and Secure Messaging that represented digital patient engagement. On the basis of Delphi theory, our in-depth interviews, and the theoretical model, we established success criteria to guide inclusion in the preliminary measures, process goals, and consensus criteria for each round of the Delphi process.

For the Delphi panel, we convened 12 national (US) eHealth experts, who were principally physicians. Our Delphi protocol, conducted in March-October 2013, involved three rounds of panelists’ independent rating of the measures; panelists submitted their ratings through a secure online questionnaire, enabling the research team to score and analyze the results while maintaining panelist anonymity to all but the researchers. To ensure that the process would ultimately yield measures of digital patient engagement, and following procedures established in prior Delphi panel studies, we asked panelists to rate the importance of each proposed measure on an 11-point integer scale, ranging from −5 (strongly disagree) to +5 (strongly agree). Our objective criteria enabled us to accept or reject a measure after each round, or to revise it for retesting in the next round. For acceptance, a measure was required to meet all three of the following conditions: (1) median score ≥+3; (2) interquartile range (IQR) ≤2; and (3) ≤1 outliers (defined as a score of >1.5×IQR from the 25th or 75th percentile). For example, consider the following 12 panelists’ scores for one measure: 0, 0, 1, 2, 3, 3, 3, 3, 4, 4, 4, 5. The median score is 3, satisfying condition (1). The IQR is 2, satisfying condition (2). There are no outliers, that is, no scores lower than −1 and no scores greater than 7 (the latter not being a possible value, given the −5 to +5 scale), thus satisfying condition (3). Therefore, this measure would be accepted and not considered further in subsequent panel iterations. For revision and retesting (in a subsequent round), a measure was required to meet two of the three conditions. If it failed to meet at least two conditions, it was rejected.

## Results

All 12 Delphi expert panel members completed the study’s three rounds of measures rating. The final candidate measures of digital patient engagement comprised two similar but separate sets: 58 measures for Secure Messaging and 71 measures for MHV/Blue Button, where Antecedents represented 20 comparable measures for both functions, Processes represented 32 Secure Messaging and 45 MHV/Blue Button measures, and Outcomes represented six comparable measures for Secure Messaging and MHV/Blue Button.

As an example of how the Delphi panel results were used to include or exclude measures, Table 1 shows the four digital patient engagement outcome measures. For each of these four outcome measures, acceptance was achieved in the third and final round of the Panel’s deliberations. Among the four measures, there was only one outlier panel member rating, reflecting high level of agreement on the value of these items in measuring digital patient engagement.

**Table 1:** Digital patient engagement outcome measures—Delphi panel statistics. Secure Messaging = first statistic and MyHealtheVet /Blue Button = second statistic, reported (in #/# format).

Measure: patient engagement	Accept round	IQR^a^	Outlier	Median	Mean	SD^b^
I have all the information I need to manage my health and health care.	3	1.5/2.0	1/0	3.5/4.0	3.1/4.0	2.3/0.9
I am confident in working with my VA^c^health care team to manage my health and health care.	3	2.0/1.5	0/0	3.0/3.0	3.7/3.6	1.0/0.8
I feel in control of my health and health care (such as taking part in decisions or following through on any medication, treatment, or health routine).	3	1.5/2.0	0/0	4.0/4.0	3.5/3.6	1.4/1.5
I am able to achieve my long-term health and health care goals (such as being self-reliant, living longer and better, or knowing that my family and friends can depend on me).	3	2.0/2.0	0/0	3.5/3.5	3.5/3.6	1.5/1.6

^a^IQR: interquartile range.

^b^SD: standard deviation.

^c^VA: US Department of Veterans Affairs.

## Discussion

### Principal Findings

Measuring how patients use HIT is a high priority for US health care. Nevertheless, existing meaningful use measurements have focused on clinicians’ use of technology with few guidelines for patient adoption and use. While various scales have emerged to assess patient engagement and satisfaction with health care, none has combined patients’ affinity for the technology with patients’ trust in their relationship with clinicians, in person and online, to demonstrate how these variables influence digital patient engagement with their health and health care.

In this national expert panel study using a modified Delphi technique, we consolidated and refined two complementary versions of candidate measures to assess patients’ use of VA's My HealtheVet patient portal, one set of measures for its Secure Messaging feature and another for its Blue Button personal health record and other MHV tools, with the potential for gauging digital patient engagement.

This study offers a number of strengths and innovations. Guided by a theory-based framework, we first developed and refined a new four-item digital patient engagement outcome measure based on Rogers’ “Diffusion of Innovation” model [22]. That is, the four new measures we developed (ie, awareness and understanding, skills and confidence, trial and regular use, and use loyalty and recommendation) map roughly to Rogers’ four stages of diffusion of innovation, namely knowledge, persuasion, decision, and confirmation. Grounding our measures in Rogers’ framework was suggested by and affirmed by the Delphi panel. The four-item digital patient engagement measures also align with Hibbard et al’s patient activation measures [23], paraphrased as (1) belief in having an active role in care, (2) confidence and knowledge to take action, (3) taking action, and (4) staying the course under stress.

As an innovation, the Delphi panel led us to introduce the novel process dimension of patient online care quality. This measure reflects the quality of the interaction of users with the technology.

We strengthened the content validity of the measures, a principal goal of using the Delphi technique, by assuring 100% participation of our content experts across the three rating rounds. To mitigate threats to external validity, such as selection bias, we selected our national experts to reflect a broad perspective of various health systems, diverse patient user groups, and an array of patient portal architectures and features. Generalizability of results also benefited from the inclusion of the expert views of the key informants. We reduced Delphi panel process threats by (1) making study goals and procedural guidelines clear at the start, (2) presenting a fair and transparent rating process with timely survey administration and response to panelist questions, and (3) extending full consideration and discussion on any dissenting opinions by panelists.

### Limitations

A potential limitation of the study is that we employed a panel of experts, rather than patients themselves, to refine and prioritize the measures for digital patient engagement. We chose our approach because we considered the tasks of measure selection to require not only familiarity with the patient portal tools and their role in health care delivery but also a comfort level with the process of questionnaire item development and measurement scales. To ensure that the measures developed in this study truly reflect digital patient engagement, they must be validated among a population of patients who are users of the portal and its functions.

### Conclusions

Establishing a valid and reliable scale is the first step to measuring digital patient engagement and its role in health and health care quality, outcomes, and effective, efficient implementation by health care providers and health care systems. This study yielded a robust set of candidate measures of what Veterans value in Blue Button and Secure Messaging. These measures and the scales they constitute can thus be tested empirically to examine their psychometric properties and may ultimately be used in measuring the extent to which patient portals and other patient-facing technologies can engage patients in their health care.

## Abbreviations

HIT: health information technology
IQR: Interquartile range
MHV: MyHealtheVet
VA: US Department of Veterans Affairs
